# Acknowledgement to reviewers

**DOI:** 10.1530/JOE-268-A1

**Published:** 2026-01-21

The editorial board wishes to thank the following individuals who have helped to review manuscripts for *Journal of Endocrinology* during 2025. Their assistance is gratefully acknowledged.

**Table:** 

D H Abbott
R D Abbott
M M I Abdalla
M Abdellatif
M Abdelmalek
N Aberdein
A Abrunhosa
G A Abruzzese
M J Abuzzahab
A Acosta
N Acs
A Adamska
U R Addi
A Aflatounian
A Agil
C Aguayo-Mazzucato
N S Ahanchi
K Ahmad
M H Akhter
F Aktoz
R K Akyea
A Akyol
B Albert
M Alcala
S Aleksandric
A Aleksova
M Alfatama
C M Alfieri
H Ali
H Ali Nagi Al-Jamal
E Alipoor
M Alkafafy
A A Alli
R Allikmets
E Allott
M Allouh
L Almeida
M Y Alsagaff
M Al-Shabrawey
F Althammer
Z Altın
J Altirriba
Ö Altun
N Aluru
S Alzahrani
A Aman
J Ambroszkiewicz
J P d A Amorim
X An
Y An
P Anagnostis
R A Anderson
B Z Andrade
R Andrew
T Andrew
S Angioni
B Anguiano
G Annibalini
A Ansarullah
U Anyanwagu
O Anzala
N M Appelman-Dijkstra
G Aragones
E J Arany
E Araújo
L M Armelin-Correa
A I Arroba
M Arslan
G E Arteel
M Arumugam
P Arvan
K R Aseer
T Ask
S Aslani
Y Aso
T L Assimes
T S Assmann
P Atanes
E I Atli
M Atoum
A Aureli
A K Azemi
D Azzolino
M Baack
A Y Babenko
L A Bach
J Badimon
V L Bae-Jump
N K Bagri
X Bai
S Bakhashab
A Bala
J Balibrea
D Balogh
A Banerjee
E Banke
K K Bansal
M Bansal
M Barat
J Barrera-Chimal
M L Barreto-Chaves
C Barth
B Bartosch
L Batista
M-L Batista Jr
T Baumert
N M Bazhan
L Beber
B R Beck
K M Beekman
F Beheshti
A B Behrooz
D Bellavia
S Bellusci
C Benavides-Reyes
C Benezech
B Bengsch
A Benrick
A Beqqali
C T Bergamaschi
S Berhane
D J Bernard
J R Bernardi
N Bernardini
S Bernasconi
P Bernstein
A Berthon
G Besutti
G Bewick
S Bhagra
J K Bhardwaj
M A Bhat
R Bhatnager
M Bhattacharya
S K Bhattamisra
V Bianconi
J P Bidwell
M Biehl
A Bilberg
V Bimonte
O Binah
A Binda
L Bindels
E Binder
D Bi-Rong
C Blanchard
K Blaslov
H Blau
S Bleich
C Bleilevens
F F Bloise
S Blouin
B Blumberg
S Blüml
L D Bobermin
L Bode
S Bodine
A Boelen
I Bogacka
S T Bond
I L P Bonfante
M L Bonfleur
C d S Borges
J Boucher
J Bouitbir
P-M Bouloux
S Bouret
A Boutin
L Bouys
J Bover
C N Boyle
L Branco
J Brandts
S Breit
K Breitkopf-Heinlein
E Brennan
S C Brennan
D Briana
L Brisson
J Brito
G Brock
S-A Brown
M Bruce
A Brüel
E Bruinstroop
N Brunetti-Pierri
P J Brunton
C Bucolo
M Budzinska
C Buechler
P Buettner
C Buffet
N Buisine
A Bustamam
A Butler
L M Butler
A Byford
A Cabrera-Pastor
J Cai
A Caldwell
J T Caldwell
R B Caldwell
L Calivarathan
M Calizon
C Callegari
R Callister
M Campbell
R Campbell
S Camper
F Campolo
E Camtosun
A A Canales-Aguirre
P D Cani
G Cantini
X Cao
Y Capodanno
J M Carli
E Carrillo
D Carvalho
I Casimiro
A F Castillo
C A Castro
A Catalano
E Cavalier
W P Cawthorn
A Cecere
G Cediel
V M Ceglarek
J Cen
R Chakaroun
S Chakrabarti
S-Y Chan
H Chang-Quan
K E Chapman
M Charlton
C Chauveau
A H Chaves-Neto
K Chechi
C Chen
C-H Chen
C-M Chen
G Chen
H Chen
N Chen
Q Chen
S L Chen
W Chen
Y Chen
Y-J Chen
Z Chen
J-C Cheng
L Cheng
S Cheng
T-Y Cheng
C Chenu
L Y M Cheung
W-H Cheung
K H Chhabra
J Chi
J Chiang
H Chitme
C-L Chng
K Y Cho
J-Y Choi
Y K Choi
A Chowdhary
M Christian
J S Christiansen
L Chu
R M Chugh
A Ciccodicola
K Cieplinska
D E C Cintra
M R Ciriolo
A Cittadini
M Claesson
M Claret
B J Clark
G Clarke
C Clarkin
K K B Clemmensen
M S Clerget-Froidevaux
V Coelho
A Cohen
T Cole
G Colleluori
S Collins
S Colucci
F V Comim
S Conde
A J Conley
D Contartese
P M Contreras
S H Cook
P S Cooke
F Cordido
J Cordoba-Chacon
J Cornish
T Corson
S Corvera
T Coskun
M L Costa
I Couto-Gonzalez
A Crino
A B Crujeiras-Martínez
J S M Cuffe
F Cui
R Cunningham
M Czlapka-Matyasik
L Da Dalt
A S R da Silva
P Dabla
A Daci
M Dalamaga
K T Dalen
L T Dalgaard
A Daliry
M K Das
S Dasgupta
D Davis
M de Courten
T de Haan
L P de Moura
D de Paula Faria
L de Sanctis
S M C De Sousa
F De Vadder
T C Delgado
M Delvecchio
F J DeMayo
O Demir
L den Hartigh
H den Ruijter
T M Dencic
Y Deng
S Deniz
F Denk
A Dennis
M Dentice
K V Derkach
M Derrien
T Derrien
E Desroziers
P Devi Nivean
E Di Cicco
G Di Dalmazi
G Dibernardo
S Diciotti
A M Diehl
N Dierichs
W Dik
G K Dimitriadis
I Dimova
A Djordjevic
P Dobrowolski
A Dobrzyń
J A Doering
C L Doig
M T Donato
S Dong
Y Dong
Z Dong
M Dorfman
R S Douglas
K M A Dreijerink
X Du
Y Du
R G Duft
J Duhault
M Dulac
R Dumbell
D Dumesic
R Duncan
W C Duncan
J Dupont
G Duque
K Eade
P Ebeling
L Edwards
H T El-Bassyouni
F Elefteriou
C El-Hadad
L Eliasson
N El-Khazragy
L Ellsworth
M Elschot
S Emamgholipour
M Emoto
R T Enos
F Ercan
P L Eriksen
A Eroglu
L Escriva
M Eslam
U R Essien
E A Esteves
A Estienne
E A Evans
K J Evason
Y Ezura
M Fabbraio
B Fam
B Fam White
X Fan
L Fang
G Fanni
G Farello
G Farmer
C Farquharson
A Fattahi
S Fatumo
D J Fazakerley
P K Fazeli
I Fecka
S Feijoo-Bandin
A Feraco
R Fernandes
M Fernández
M Fernandez-Galilea
J M Fernández-Real
S Ferosekhan
A C F Ferreira
S T Ferreira
A Ferreira-Hermosillo
J Ferrer
C J Ferro
W T Festuccia
J Fielitz
A Fierabracci
T Filardi
B N Finck
D Fintini
J Fish
G Fisher
J Fisher
J Flannick
J Flaws
I Fonseca
T Fonseca
K Forbes
C Formichi
P Formisano
S Forte
R S Fortunato
M F Fountaine
L Fozzatti
L Fraga
M C Fragoso
V Francisco
P Franks
R Freahy
H Freisling
W Freitas Jr
L C Freitas-Lima
A Freund
M Friedlander
S L Friedman
L Frittitta
J Frokiaer
F Fu
D Fuehrer
D Führer-Sakel
Y Fujitani
S Fukumoto
A Fukushima
P J Fuller
C L M Furtado
S Furukawa
J Furuzawa-Carballeda
V Galbiati
J Galitzky
J Gallo
S Galmes
D Galuska
P Gamez
V Ganapathy
M A Ganie
M Gantner
D Gao
F Gao
H Gao
T Gao
Y Gao
C Gar
A Garbe
M Garcia-Arranz
A Gartland
L Gasberg
H Gatterer
D Gatti
V A Gault
A Gaur
M Gawałko
R F Geddes
B Geloneze
R Gelpi
G Gembillo
T Geng
M Gericke
G Gervasini
S Gharanei
A Giaccari
A Giacco
D A Gibson
A Gilbertsen
R B Gilchrist
S Gilley
C M Girgis
N Giribabu
G Gittes
L C Giudice
A Giustina
D Glass
D Glintborg
G Gobe
L Goense
P Goes
K Gomes
N Gomes
R A Gomez-Diaz
C E Gomez-Sanchez
E P Gomez-Sanchez
Z Gong
J Gonzalez
P Gonzalez-Muniesa
C M Gorvin
T Goto
G Gourley
L Gouveia
K Gowdy
L Graaff
I Grabowska
J B Graceli
C Graf
N Gray
M Green
N P Greene
F M Gribble
H R Griffiths
W Griffiths
J J Gross
G Grugni
D Gruson
G Gu
M Guarino
B Guigas
T Guilarte
G Guillemin
H Guillou
Z Gunendi
G Guney
J H Gunter
S Guo
F Guppy
D Gupta
S Gupta
S K Gupta
O Guralp
S Gutierrez
K Gutowska
T S Guvenc
O Guzeloglu-Kayisli
P T Ha
M Habibi
A Hadjichambi
M Haffner-Luntzer
M Haid
M Hamrick
J Hamulka
A Han
C Han
V Han
Y Han
N Hancerliogullari
M Hany
M Haque
M Harada
J Hardesty
J Harreiter
C A Harrison
J Z Harvey
A Hasib
K Hata
J R Hawse
B He
H He
L He
Y He
C P Hedges
G H Heeba
J T Heiker
U Heilmeier
A Henney
A Herranen
P Herrera
L Herrero
M Herrmann
E Hesse
E V S Hessel
A Hevener
M Hewison
W Hildebrandt
C Hill
W D Hill
C Hilton
T Hinds
K Hirasaka
Y Hirota
J Ho
K K Y Ho
Y R Ho
S Hocking
L Hoffman
C Hoffmann
D B Hoffmann
M F Hogan
B Hoh
A Hokken
R M Holden
G J Holloway
H Holthofer
N Hong
S Hong
Y Hong
M S Hossain
F Hosseini-Esfahani
S Hourigan
A Hoy
L Hoyles
S-M Hsia
B-G Hsu
M G Hu
T Huan
K Huanbutta
C-C J Huang
S-W Huang
W Huang
Z Huang
J Huertas
J Hughes
I T Huhtaniemi
M O Huising
M Hullar
K Hummitzsch
T J Hureau
J Hurley
L Hussain
M Hussein
K Hutt
C Huttenhower
A Huxtable
T J Hydes
K Ibos
I A Ibrahim
D Ilatovskaya
I Ilias
Y Imai
H A Ingraham
M R Insenser
N Irwin
J Ivy
T Iwasa
A Iwase
P Jacobs
A J Jalkanen
S Jana
U Janssens
T Jarosz
N Javeed
P Jaworski
S Jeddi
C Jenkinson
K Ji
Q Jia
D Jiang
H Jiang
N Jiang
Z-L Jie
W Jin
J Jones
E Jönsson
L Jorm
P-A Jose
M Jovani
P Joyce
J L Juengel
H-S Jun
H-W Jung
W F Junior
N Kaftan
H Kahleova
C R Kahn
H Kainulainen
H Kaji
E Kalaitzoglou
A Kalsbeek
I Kanakis
J Kanaley
S Kanda
J Kang
K Kaňková
A Kantarci
G Kapravelou
O F Karatas
R Karne
S Karvinen
A Kasajima
M Kashii
M Kassem
E Kassi
T Kataoka
G Kaur
J Kaur
S Kaur
T Kawahara
N Kawao
M Kaynak
D Kayo
M Kazim
S Keipert
M Keller
M Kelley
M D Kendig
B KenKnight
G F Kerkhof
H L Kerr
K Kerschan-Schindl
R Khalaf
M Khalaj-Kondori
S A Kharroubi
A Khathi
B Khoo
E M Kileel
H-S Kim
I Kim
J Kim
S Kim
Y Kim
T Kimura
R D Kineman
R Kinscherf
A Kiss
M Kittel
C Klemens
M C Klymiuk
K Knapczyk-Stwora
J Knez
F Ko
H Kobayashi
Y Kobayashi
S B Koca
S Koçak
J-T Koh
N Koitabashi
Z Kokkalis
A Kokkinos
M Koland
T Kolettis
H Komaba
M Komrakova
R Kon
E Kondo
B Kong
S Kooijman
N Koonrungsesomboon
L L Koorneef
K Kopeva
J Kopp
T Korevaar
J-W Kornfeld
K Kosmac
E Kostallari
K Kotsa
T Koufakis
M Kouvari
K Kowalczyk
M Kowalewski
A Kowalska
R Kowluru
M Koziolek
K Koźniewski
H Krakowski-Rosen
K Krambeck
K Krause
N A J Krentz
C Krischek
A Krishna
V Kronsten
J Kroon
M J Kucharczyk
J C Kuchler
P Kudtarkar
V Kumar
A E Kurylowicz
S Kushwaha
J Kusuyama
Y Kuthati
S Kwak
S E la Fleur
S Lagarrigue
M Laloraya
A Lama
B LaMarca
C B Lambalk
J W Lampe
J Lang
P Laranjeira
J L Larson-Casey
S Larsson
J Latorre
V Laubach
M Laudes
R Lavado
H A LaVoie
B J Lawrence
A Lázaro
C Lazzaretti
I J Lean
V Leandro-Merhi
G Leanza
C Lebrilla
B Leckaczernik
B-C Lee
D H Lee
J H Lee
S Lee
V Lee
Y Lee
P Leete
M Lehrke
Z Lei
E C R Leonel
F Leonetti
I K Lesna
H Leung
P S Leung
G Levate
D F Levey
J Levita
A Levy
E Lewin
M Lewis
B Li
G Li
H Li
J Li
L Li
M Li
N Li
Q Li
X Li
X-y Li
Z Li
V Liakopoulos
A Liang
R Liang
C-D Liao
H Libouban
S A Liddelow
C-J Lin
K E Lines
B Ling
C Ling
G Lingling
A Linnemann
F Lira
W Lisik
S E Little-Letsinger
A Liu
B-P Liu
D Liu
F Liu
G-J Liu
H Liu
H-y Liu
K Liu
M Liu
S Liu
X Liu
Z Liu
A Lodberg
C Logsdon
G Lombardi
L Longhitano
P Lopez
S Lopez-Enriquez
M Lorentzon
M V Lourenco
B Lu
C Lu
S Lu
W Lu
Y W Lu
Y Luan
M Lucarelli
R Lucas
M Luconi
L Lundby-Christensen
L Luo
T Luo
K Lutfy
P Luukkonen
Q Lv
W Lynch
F Lynn
H Ma
N Ma
P Ma
X Ma
G Mabilleau
S Macari
M Macaskill
C K Macgowan
R M B Maciel
K Macleod
G J Macpherson
B Macq
E Maddaloni
A A Madhoun
Y Maejima
D Mafra
C Magalhaes
C Magnan
A A Mahabadi
A Mahfouz
A M Mahmoud
M Mahoney
A Mahor
R Mak
E N Makarova
M Malaguarnera
H Malekinejad
C A A Malheiros
C Malvestutto
M Mamenko
H Mannell
A Mansoori
P Mantuano
C Mantzoros
L Manuel-Apolinar
G Many
A Maranha
K P Maremanda
M Marinò
T P Markovic
K Marra
B Marshall
J G Marshall
K J M Martin
L J Martin
J A Martinez
M Massaro
V d P Mathew
M Matos
J S Mattick
D Mattson
H Maude
G Maurizio
S Mayerl
L McCabe
C McCarthy
C J McClain
E V McCloskey
M E McGee-Lawrence
A Meakin
L B Meakin
A Megía
W Mehal
R Mehta
H Mei
O C Meijer
A Meiliana
A Meissner
K Mekeel
P Melby
R C Melcangi
D Melo
F Menconi
A E Mendham
K Meredith-Jones
B Meriga
C Metallo
K Mezian
T Mezza
S Mhaouty-Kodja
D Miao
A Miatmoko
Z Michailidou
M-L Michel
M M Michelsen
J Micieli
B S Miller
K K Miller
J Millman
S Milne
L Y Milovanova
X Ming Shi
L Minuzzi
L Miranda-Alves
J Mitchell
T Mitic
H Mitsutake
J Miura
S Miura
M Mizgier
A Mizokami
N Mody
L C Moeller
S Mohan
T Moholdt
M Mokhtar
A Mokhtarzadeh
P Molina
I Molnar
M Momin
S Monga
J Monks
D G Monroe
A Morales
S Morano
I Mordi
M L Morieri
L Morin-Papunen
Y Morishita
M Morris
M C Morrison
S Morrisson
A Motala
K J Motyl
D Moyes
R M A Moyses
J Mozziconacci
L Mu
J W Mueller
S Mukherjee
C Muller
E Murata
S Muroya
K G Murphy
F Muscatelli
A S Muscher-Banse
N Musco
M Musella
T Musholt
M Myers
X Nabi
M Nagao
R Najafi
M G Nanna
S P Narayanan
M Nascimento
G Nassis
R Natarajan
M Nathanson
E Nebot
C H Nery Costa
I C Nettore
M S Y Ng
X Ni
G Nie
P Nighot
L Nigi
M Nixon
V U Nna
H Noguchi
T Nomikos
C Noordam
D Norata
B Noszczyk
M Notsu
R Nourizadeh-Lillabadi
B Nowak
F Nualart
C Nucera
E Nudleman
M T Nunes
M O’Sullivan
P O’Connor
S O’Dell
A O’Donohue
C-M Oh
F O’Harte
C Ohlsson
Y Oka
H Okada
K Okada
P N Okechukwu
M R Oliveira
A Olivera
M-M Ollila
K Omote
Y Ono
J Opydo-Szymaczek
I Oriss
L Orlando
F J Ortega
T Oshima
J Osorio
Ó Osorio-Conles
S E Ozanne
A C Ozay
O Ozmen
V Padmanabhan
A Palmquist
X Pan
G Panahi
P Pandolfo
F Pani
T Paolucci
D Papandreou
S-Y Park
V Parker
E E Parkes
D Pascut
L Pasquali
R Patarrao
O Patey
S H Patlolla
N Patrova
A Patruno
I Paul
A Pavlík
M Pazianas
E Peckham
R Pecori
R Peila
J Peng
F Pereira
R M Pereira
S Pereira
J Pereira das neves
A R Perez
A V Perkins
J Persson
S Peters
M Petkovic
S Petousis
H L Petrick
M Phan
A Philippou
E Piccinin
M Piccioto
N Pillon
Y Ping
L Pisani
S Piscuoglio
A Plagemann
L Plotkin
C Poderoso
V Poitout
A Pollard
G Ponti
E Ponzini
K Pool
L Poole
O Popa-Velea
Y Popov
J E Posey
F Poulat
P Pramyothin
B Predieri
E Prescott
E K Pressman
G Prins
H-B Pu
J Qian
Q Qiu
D Y Quansah
E D Queathem
J S Quist
S-G Ra
D Rachon
E Radmanesh
R Raeman
A Rafacho
S Rafiq
F Ragusa
W Rainey
A Rak
L Ramalingam
B Raman
S K Ramchand
J L Ramirez-GarciaLuna
D Ramiro-Cortijo
N Ramkumar
R V Ramos
P Ramos-Ibeas
S Ramos-Romero
G Rampazzo
L Raposo
M Rauner
D W Ray
Y Raykov
C C Real
R S Rector
D A Rees
D Regazzo
J Rege
C Regina Nogueira
C Rehfeldt
I L H Reichert
D Reid
F Reis
L T Reiter
A Relling
A Relogio
P C N Rensen
T Rentz
A Ribeiro
G Ribeiro
R Ribeiro
R A Ribeiro
J Ribot
L Riou
A A Rizvi
D Robinson
C N Robson
R J Rodgers
L Rodrigues
R Rodriguez-Calvo
T Rodriguez-Calvo
N Rohmann
M Rohrberg
D G Romero
C Roncal
X Rong
J F Roos
J C Rosa-Neto
S D Rosen
D Rosendo-Silva
M Rossini
C L Roth
M Roumain
C Rovere-Jovene
M Rovira
H Roy
V K Roy
P J Rozance
T Ruiz
S B Rulli
J Sacramento
E Sadowska-Krepa
M S Sagmeister
T Sallam
N Salleh
J Salles
A Salvatoni
S Samarajiwa
A Samidurai
M Sanchez-Garrido
D Sanchez-Infantes
R Sanchez-Varo
R Sancho
J Sancho-Pelluz
M J Sandoval
C Sanford
M Sang
I Santin
D Santos
V Sardao
A Sati
I Satman
Y Sato
E Saw
C M Scannell
S Scarcella
B Scazzocchio
C P Schaaf
P E Scherrer
J Schiavi-Tritz
M P Schneider
S Schon
J Schoonmaker
J Schroder
T Schroeder
A Schuermann
R Schulz
T W Schwartz
R Scicali
D Scott
S A Sebastian
R J Seeley
S Sehmisch
Y Seino
E Seki
N Selvamurugan
C Sena
K Seretis
A Sertedaki
E Shakhtshneider
K Shankar
X Shao
M H Sharawy
E Sharifi
A D Sharma
K Sharma
S Sharma
N Sharon
N Sharrack
M S A Sheikh
C-a Shen
J Shen
S-C Shen
Y Shen
J-H Shi
P Shi
X Shi
Y Shi
V M Shipulin
B Shirouchi
S Shoaie
A Shoemaker
M Shukla
G Shulman
M Si
V Sidarala
A Siltari
A C Silva
F Silva
J F Silva
R D Silva
C R Sims
E K Sims
K Singer
A Singh
D D Singh
P Singh
S Singh
S Singhal
I Sinha-Hikim
B S Siqueira
M Skrzypski
A Sliwinska
A Smieszek
T J Smith
U Smith
Z Smit-McBride
P B F Soares
S Soderling
S Soleimanpour
A Solomon
K Somnay
J S Son
E Sondergaard
X Song
S Sonkusare
A Sørensen
M Soresi
B Soria
E Sorianello
A Soto
A Soto-Gutiérrez
Z Souri
R A L Sousa
P M R Sousa e Silva
J Sowers
J M Spaeth
K Spalding
J Speed
B Spiegelman
S Srinivasan
C Sriphrapradang
R Srivastava
K Staines
A Stamatikos
K I Stanford
T Stanley
F Staud
A F Stewart
C Stewart
R H Stimson
L Stojanovski
S S Stojilkovic
T Stompor
N C A Strock
T V Strong
E K Stuermer
K M Stuermer
M Styner
J Styrke
H E Suhrs
V Sukhorukov
O A Sukocheva
S Suman
B Sun
J Sun
Q Sun
R Sun
W Sun
X Sun
H-K Sung
R Sungthong
F Suriano
P L Surico
A Suzuki
T Svingen
F Syed
M Szczuko
W Szkróbka
H Q Ta
I Tabas
H Taenaka
A Taha
N Tahani
R Taliyan
W Tan
T D Tanaka
J Tang
Q Tang
X Tang
L Tao
G Targher
T Taskin
D A Tata
M Tauber
P Taylor
A Teixeira
M Tena-Sempere
M Tencerova
Y Terauchi
J Tessem
F N Tessler
R Thapa
P Tharaux
K Thirumurugan
P Thomsen
F Thorsteindottir
M Thuzar
L Tian
C Toda
L Tolosa
M L Tomasi
E Tomaszewska
F Tona
H Tong
T Tong
W Tong
A Topiwala
E Topol
B Torensma
G Torres
I Torre-Villalvazo
A S Torsoni
M Toumba
M Tran
A Travaglino
J J Tremblay
S-J Tsai
A Tsuji-Hosokawa
T Tu
J Tuckermann
Y-C Tung
S Turgut
A Turnell
M C Turner
T L Tyasi
S J Tye
C R Tyler
H Ueland
H Uhlenhaut
A B Upaganlawar
A Uruska
A N Vadakkepushpakath
F Vahid
E Vale-Fernandes
M Vallée
B W van der Kolk
A H van der Spek
L van Grunsven
D van Raalte
L Varela
B Vastrad
K L Vaughan
R Vaughan
D Vauzour
A Veiga-Lopez
S K Vemula
B Verchere
J M Verdes
J Verdu-Soriano
A Verma
H Verma
C D Verrico
J F Vettorazzi
M Viana
S Viana
C Viguie
S Vilarinho
E Villalobos
A Villani
A Virmani
S Vitale
D Vlastos
D Vohora
D Vojnović Milutinović
B J Waddell
M Waddingham
R Wadgaonkar
S M Wajner
J M Wallace
R Wallace
K A Walters
L Wan
X Wan
P L Wander
C Wang
C C Wang
D Wang
H Wang
J Wang
L-J Wang
S Wang
W Wang
X Wang
X-L Wang
Y Wang
Z Wang
S K Wangnoo
U Wankhade
G R Warner
E F Wasfey
M Wąsowski
N Wathoni
D J Waxman
J Webber
A Weber
R Wei
Z Wei
L S Weinstein
R Weiskirchen
J M Weitzel
R J Welly
H Wen
E Wender-Ozegowska
C Wenzek
S Wesolowski
M B Wheeler
C A White
S Wiberg
H S Willenberg
H Willenbring
E N Wilson
I S Winer
N C Winn
G Woldeamanuel
S K Wong
S Wray
D Wright
J Wright
A Wu
C-M Wu
D Wu
H Wu
L Wu
S Wu
W-K Wu
W-L Wu
Y Wu
Y-L Wu
S Wueest
B Wynne
D Xia
P Xia
M Xiao
Y Xiao
C Xie
H Xie
W Xie
C Xu
D-X Xu
G Xu
J Xu
L Xu
R Xu
X Xu
Y Xu
Y Xue
D Yabe
S Yadav
C Yallampalli
K Yamada
K Yamanishi
T Yamauchi
L Yammine
X Yan
L L Yanes Cardozo
C Yang
H Yang
J Yang
Q Yang
X Yang
X-W Yang
J Yanovski
A Yanuar
H Yao
M Yasir
M Yates
L Ye
B Yegen
J Yi
Y Yi
L Yin
Y Yin
H Yong
J S Yoon
S H Yoon
B Young
J Yu
L Yu
X Yu
X-J Yu
H Yuan
Z Yue
K Yuen
J Zanker
P Zaslansky
E Zemanick
G Zeng
M Zeppieri
Q Zhai
C Zhang
D Zhang
J Zhang
R Zhang
W Zhang
X Zhang
Y Zhang
Y Q Zhang
D Zhao
H Zhao
L Zhao
B Zheng
H Zheng
H Zhou
Z-B Zhou
C Zhu
M Zhu
S Zhu
M Zielinska-Pukos
J Zigman
D B Zoccal
X Zong
X Zou
P Zouhar
S Zraika
C Zuurbier

